# Insights on exclusive breastfeeding norms in Kinshasa: findings from a qualitative study

**DOI:** 10.1186/s12884-020-03273-4

**Published:** 2020-10-06

**Authors:** Francine E. Wood, Anastasia J. Gage, Dieudonné Bidashimwa

**Affiliations:** 1 School of Public Health and Tropical Medicine, Global Community Health and Behavioral Sciences, Tulane University, 1440 Canal Street, New Orleans, USA; 2 School of Public Health and Tropical Medicine, Health Policy and Management, Tulane University, 1440 Canal Street, New Orleans, USA

**Keywords:** Breastfeeding, Qualitative methods, Breastfeeding barriers, Social norms, Qualitative methods, First-time mothers

## Abstract

**Background:**

For optimal growth and development, the World Health Organization recommends that children be exclusively breastfed for the first 6 months of life. However, according to the nationally-representative 2013–2014 Demographic and Health Survey, under 50% of babies in the Democratic Republic of Congo are exclusively breastfed. Although breastfeeding was common in the capital city of Kinshasa, one in five newborns received alternatives to breastmilk during the first 3 days of life. This analysis aimed to identify social norms influencing exclusive breastfeeding, the role of a young first-time mother’s (FTM’s) social network for her choice to exclusively breastfeed, and perceived social sanctions associated with breastfeeding practices in Kinshasa.

**Methods:**

The qualitative analysis was based on a vignette presented during 14 focus group discussions, with a purposively selected sample (*n* = 162) of FTMs age 15–24, and the male partners, mothers and mothers-in-law of FTMs age 15–24 in three health zones in Kinshasa in 2017. Thematic content analysis was performed to identify concepts and patterns in the participants’ discussions.

**Results:**

Overall, community norms were not supportive of exclusive breastfeeding. The main barriers to exclusive breastfeeding were the belief held by most FTMs that exclusive breastfeeding was an uncommon practice; the desire to avoid negative sanctions such as name-calling and mockery for refusal to give babies water in the first 6 months of life; the desire to please key members of their social networks, specifically their mothers and friends, by doing what these influencers expected or preferred them to do; FTMs’ own lack of experience with infant feeding; and trust placed in their mothers and friends.

**Conclusion:**

Social norms can be maintained by the belief about what others do, perceived expectations about what individuals ought to do, the negative sanctions they can face and their preference to conform to social expectations. Thus, addressing cultural beliefs and targeting sensitization efforts to key influencers that provide support to FTMs are needed to promote exclusive breastfeeding in Kinshasa. In doing so, strategies should address the barriers to exclusive breastfeeding including related misconceptions, and improve FTMs’ self-efficacy to overcome the influence of others.

## Background

The World Health Organization (WHO) and the United Nations Children’s Fund (UNICEF) recommend that newborns be put to the breast within the first hour of birth and, for optimal growth and development, that children be exclusively breastfed for the first 6 months of life, during which they must not be given other foods or liquids, including water. However, only 40% of babies worldwide [1] and 48% of those in the Democratic Republic of Congo (DRC) are exclusively breastfed [2]. Despite breastfeeding being near-universal in the capital city of Kinshasa, with 98% of children born in the past 2 years being ever breastfed, one in five (20.8%) newborns received something other than human milk during the first 3 days of life before being breastfed [2]. Suboptimal breastfeeding practices have been associated with increased risk of gastrointestinal problems, increased risk of dying from respiratory and diarrheal diseases, and with more than 800,000 child deaths annually [3, 4]. Although Kinshasa is close to achieving the 2025 target of 50% for exclusive breastfeeding, it is important to examine factors that could act as a barrier to achieving higher rates.

Studies of infant feeding practices in the DRC have noted that mothers’ poor physical health, fear of transmitting HIV to infants through breastmilk, breast health problems, and poor feeding experiences are barriers to exclusive breastfeeding among women living with HIV [5]. A mixed method analysis of suboptimal breastfeeding behaviors in Kinshasa revealed that water and porridge supplementation during the first 6 months of life was driven by a concern about the hot weather conditions and perceived human milk insufficiency, and by cultural beliefs that water must be provided in addition to breastmilk to aid digestion [6]. Similarly, in Tanzania, a mixed method study suggested that participants believed that poor breastfeeding practices were linked to beliefs that water stimulated the baby’s sucking reflex, mother’s breast milk has limited nutritional values for optimal child growth, and provision of herbs had medicinal and cultural purposes [7]. In another study, only 55% of mothers could correctly state 6 months as the recommended duration of exclusive breastfeeding [8]. Similarly, a prospective study in Kinshasa noted that mothers discontinued breastfeeding before 6 months because they had low breastfeeding knowledge, lacked confidence in their breastfeeding ability, had no plan on the duration of exclusive breastfeeding, and experienced breastfeeding problems during the first week [9]. Low breastfeeding knowledge and support skills among health care workers and high work burden, especially in agriculture, have also been identified as a barrier to optimal breastfeeding practices [8, 10–12].

Theories of individual health behavior, primarily the Integrated Behavioral Model, Social Learning Theory, Social Network Theory and Social Norm Theory, highlight the relevance of perceived social norms for an individual’s behavioral beliefs, intentions, and actions. Social Norms Theory states that much of people’s behavior is influenced by their (mis)perceptions of what other members in their social group think and do. People tend to follow a behavior if they believe that others in their social group follow it due to a desire to fit in or imitate the behavior of others [13, 14]. The social norms approach makes a distinction between descriptive norms (perceptions about what most individuals in a social group do) and injunctive norms (perceived approval or disapproval of a behavior by members of a social group). According to Social Norms Theory, an individual’s preference to conform to a social norm is conditioned on two beliefs: (1) that a sufficiently large number of people in his/her relevant social group conform to the social norm, referred to as empirical expectations; and (2) that a sufficiently large number of people in their relevant social group think he/she ought to conform to the behavior, referred to as normative expectations [15].

While social norms around health behaviors such as alcohol consumption [16], intimate partner violence [17] and condom use [18] have been extensively studied, research on exclusive breastfeeding social norms in low-income countries is limited. The objectives of this study were to: (a) examine social norms governing exclusive breastfeeding and the extent to which they mattered for a new mother’s breastfeeding choices, (b) assess the relative importance of members of a first-time mother’s (FTM’s) social network for her choice to exclusively breastfeed, and (c) explore perceived social sanctions associated with breastfeeding practices.. This analysis is based on formative research conducted in Kinshasa by the MOMENTUM project among FTMs aged 15–24 years and the male partners, mothers, and mothers-in-law of FTMs. An understanding of perceptions regarding exclusive breastfeeding is a necessary precursor for improving breastfeeding practices and promoting optimal breastfeeding as an individual and community responsibility.

## Methods

### Design

Data were derived from a brief set of questions in semi-structured focus group discussions (FGDs) that were conducted in the formative stage of the MOMENTUM project, a new gender-transformative integrated family planning and maternal and newborn health project for first-time parents in Kinshasa. The FGDs focused on exploring social norms around and barriers to the adoption of specific maternal and newborn health practices, including exclusive breastfeeding. Core constructs of the Integrated Behavioral Model and Social Norms Theory provided a framework for the data collection, analysis, and the discussion of our findings.

#### Ethical approval

This research study was reviewed and approved by the Tulane University Institutional Review Board (1112188) and the University of Kinshasa School of Public Health Ethics Committee (ESP/CE/060/2017). In addition, the confidentiality of study participants was maintained by deidentifying them and eliminating any identifying information from the FGD transcriptions.

### Setting

The qualitative study took place in three health zones of Kinshasa, the capital of the Democratic Republic of Congo: Kingasani, Lemba and Matete. These health zones were selected by the donor based on the implementation sites of a facility-based project focused on strengthening day of and after birth delivery services in selected high-volume maternities. In Kinshasa, 98% of births take place in a health facility and 97% are assisted by a skilled birth attendant [2]. However, the high institutional delivery rate does not translate into recommended maternal and neonatal health behaviors, such as exclusive breastfeeding. In the DRC, Kinshasa has the second highest rate (21%) of infants who receive liquids and food prior to breastfeeding. Fifty-one percent of children were breastfed in the first hour of delivery and 32% the day after birth. Compared to other provinces, Kinshasa has the shortest median duration of non-exclusive breastfeeding (19 months) [2].

### Sample

Participants were FTMs age 15–24 years, male partners of FTMs age 15–24, and mothers or mothers-in-law of FTM age 15–24 who lived in selected health zones. The inclusion criteria varied slightly across the different groups of participants. However, all participants had to reside in Kinshasa for at least 1 year, be fluent in Lingala, and literate at a primary school level to be able to read the consent form. FTMs were included if they were age 15–24. Male partners of a 15–24-year-old FTM were included as were mothers of unmarried 15–24-year-old FTMs and women with a son who was married to a 15–24-year-old FTM.

Participants were recruited using purposive sampling, in collaboration with the Chief Medical Officers (CMO) of the Health Zones. These individuals were contacted by a research team member to participate in the FGDs. The team member explained the nature, objectives and schedule of the FGDs, and provided them with an Invitation Coupon. Potential participants who showed up and presented the invitation coupon went through the informed consent process before participating in any research activity. Participants did not receive any payment for their participation but were reimbursed for travel expenses incurred. Recruitment and data analysis occurred concurrently until data saturation was achieved when additional focus groups discussions did not bring up new understanding of exclusive breastfeeding practices.

### Data collection

Fourteen FGDs were conducted between November and December 2017, with ten to twelve participants per focus group. The focus groups were disaggregated by age, relationship/marital status, and topic to be discussed. Discussions were held in community-based locations in Kinshasa that were easy to access, had no government or religious signage, and provided no indication that women came to participate in research activities. Each session lasted for 90 min and was audio recorded. Moderators were trained in managing emotional responses and inter-personal disagreement in a respectful way.

Written consent was obtained prior to the start of the FGD, after which those who consented to participate were asked to complete a background information sheet. Participants did not have to contribute any information they were not comfortable sharing and were told they could withdraw from the study at any point without penalty. None of the participants withdrew from the study.

### Focus group guide

**Fig. 1:**
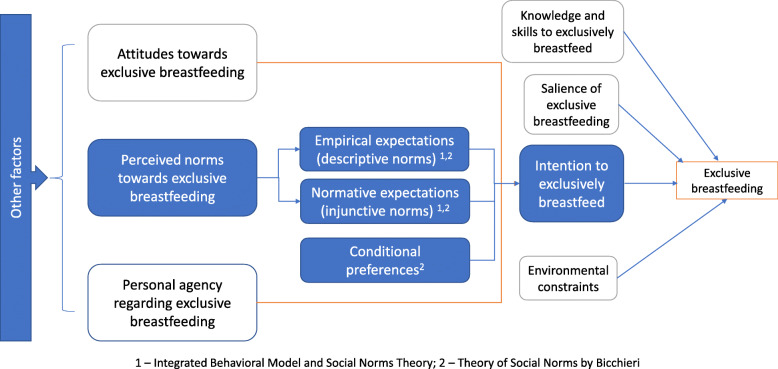
Conceptual model describing the role of social norms in exclusively breastfeeding (adapted from the Integrated Behavioral Model and Theory of Social Norms by Bicchieri). Note: The formative research questions focused on the perceived norms and conditional preferences regarding exclusive breastfeeding

The focus group guide was informed by the constructs of the Integrated Behavioral Model [19] and the Theory of Social Norms by Bicchieri [15] (Fig. 1). Information was collected on the following topics: the role of fathers and mothers in the community; decision making about breastfeeding and maternal and newborn health; forms of support that male partners provide during pregnancy; and, barriers to and enablers of gender-equitable behaviors after pregnancy. Although multiple topics were explored in the larger study, the data presented in this paper focused on a vignette about exclusive breastfeeding. The advantages of using vignettes to explore social norms are that vignettes are about fictional characters, which reduces the likelihood of social desirability bias; and are also a simple and relatable way of collecting data about social expectations [20].

#### Vignette

Focus group participants were presented with the following vignette and asked a series of questions to explore norms influencing exclusive breastfeeding.

*"I will tell you a story about Marie who is a first-time mother and is 18 years old. Marie gave birth to a healthy baby boy five days ago. Marie is practicing exclusive breastfeeding. Marie's friend, Cathy, comes to visit her. They are about the same age. Cathy strongly suggests to Marie that she should give her baby water in addition to breast milk. Cathy argues that Marie should give her baby water to drink because it is too hot and because water is needed for proper digestion of breast milk. Marie's mother who has been listening to the conversation supports Cathy's argument. Marie's mother is happy that Cathy has given Marie this advice because Marie has not been listening to her mother's advice."*

Participants were then asked a series of questions based on the pertinent constructs in the conceptual model (Fig. 1) to explore how decisions about breastfeeding are made (Table 1). Questions were created to explore normative and empirical expectations, and conditional preferences about exclusive breastfeeding. The trained moderator used open-ended questions and probing techniques to ensure that all the relevant topics were explored.

**Table 1:** Vignette Topics and Questions with the Corresponding Theoretical Constructs

Vignette topic	Construct measured	Description of the construct	Vignette question used to measure the construct
1. Empirical expectations regarding exclusive breastfeeding	Empirical expectations (descriptive norms)	What participants think others do, believe that most people do, or have seen most people do	What would most 15–24-year-old FTMs in Marie’s situation do in this situation? Would they give the newborn water in addition to breastmilk?
2. Normative expectations regarding exclusive breastfeeding	Normative expectations (injunctive norms)	What participants think others believe they ought to do	What would Cathy and most other FTMs expect Marie to do in this situation?
3. Positive and negative sanctions related to exclusive breastfeeding	Sanctions	Participants opinion of the consequences experienced as a result of deviating from the norm	If Marie decided that in spite of what Cathy says she would exclusively breastfeed her baby for the first 6 months, what would Cathy and most other FTMs who are 15–24 years old say about Marie’s decision?
4. Influence of social network	Conditional preferences	Whether normative expectations regarding breastfeeding matter or whether people’s preferences are conditional on social expectations	Would the opinions and reactions of her friends make Marie change her mind about practicing exclusive breastfeeding for 6 months?

The focus group transcripts from the study are available (in French) from the corresponding author upon request.

### Data analysis

All FGDs were conducted in French or Lingala (the most used language in Kinshasa) and transcribed in French. The choice between French or Lingala was dependent upon the FGD participants. Moderators were completely fluent in both languages. Following the completion of the FGD and the transcription, the moderators reviewed the transcriptions for accuracy, referring to the audio recordings if necessary. Transcripts were translated to English and analyzed by bilingual (French/English) speakers. NVivo 12 software (QSR International, Burlington, MA, USA) was used to organize the data and assess the emergent themes from participants’ responses. The vignette data were coded and themes concerning exclusive breastfeeding were identified. Throughout this process, the team identified quotes that captured frequent, dominant and similar responses.

## Results

### Participant characteristics

Participants consisted of 85 FTMs, 42 male partners, and 35 mothers/mothers-in-law (Table 2). Their average ages were 19.8, 24.9, and 48.6 years, respectively. Few FTMs were currently married/cohabiting (28%) compared to male partners (45%) and mothers/mothers-in-law (77%). Most participants had secondary or higher education, but mothers/mothers-in-law had substantially lower levels of education. Nine out of 10 participants were unemployed. Participants’ health zone of residence reflected the site of the FGDs. All mothers/mothers-in-law and no FTMs resided in Kingasani, while no male partners resided in Lemba.

**Table 2:** Participants Characteristics of MNH focus group participants

Characteristics	FTMs	Male Partners	Mothers/ Mothers-in-law	Total
N	85	42	35	162
Mean age (years)	19.8	24.9	48.6	27.3
Married/in union (%)	28	45	77	43
Secondary/higher education (%)	88	93	54	82
Unemployed (%)	96	86	100	94
Health zone of residence (%)				
Kingasani	0	52	100	35
Lemba	58	0	0	30
Matete	42	48	0	35

### Major themes

In all 14 FGDs, similar themes emerged. As reported below and in Table 1, the main areas of the discussion were empirical expectations, normative expectations, positive and negative sanctions and conditional preferences regarding exclusive breast feeding.

### Empirical expectations regarding breastfeeding

Participants varied in their perceptions of which breastfeeding behaviors were typical in their community. Majority of FTMs and male partners noted that most FTMs would give the newborn baby water. Mothers and mothers-in-law of FTMs had differing perceptions, most shared that FTMs would not give the new baby water. The reasons varied across the three groups.

#### Provision of water

The participants provided a myriad of reasons to support mothers’ provision of water to newborns. Participants frequently shared that some mothers provided water because of the perceived benefits of drinking water, weather conditions, their own mother’s influence, and the perceived notions that breastmilk is ‘hot’, and that water improves health outcomes for babies.

##### Myths and misconceptions

All groups of participants shared several misconceptions about the benefits of water for newborns. Many participants shared that “water is life” and babies must consume water daily. Participants believed that water gives an infant strength and energy and improves the infant’s health. This misconception led them to believe that there was no harm in providing water in addition to breast milk.

*Most new mothers aged 15-24 should follow Cathy's advice. Water must be given because water is life. While the mother is breastfeeding, give the child some water because it gives the child energy.*

*(R1: Male partner married to FTM 15–19 years)*

*Most mothers will give breast milk mixed with water because [consuming] milk alone is difficult for the newborn and can cause digestive disorders.*

*(R7: Married FTM 20–24 years)*

For some, the perceived benefits of giving water to newborns took precedence over advice received to the contrary from a health provider. One FTM shared that water had calming benefits.

*I will give water to the baby. The doctor who helped during my delivery advised me to give water to the baby at six months. I did not respect this because it was very hot, and the child was crying all the time. After giving him some water, he calmed down*.

*(R9: Unmarried FTM 15–19 years)*

Another misconception was related to weather and the temperature of breastmilk. Several of the participants explained that mothers provided water to combat the high temperature of breastmilk, which was believed to be associated with the high temperature of the air.

*As we have seen in this country, the temperature is already hot, and breast milk is hot as well. When she gives him water, it will help a lot. So, she must follow her sister’s advice.*

*(R12: Male partner of unmarried FTM 15–19 years)*

*Mary followed her mother's advice because water is a source of life. Also, it is very hot these days, the child must drink milk and water so that he is well.*

*(R3: Mother of unmarried FTM 20–24 years)*

*Most new mothers would add water to breast milk because breast milk is hot when it comes out of the breast if given alone.*

*(R11: Married FTM 20–24 years)*

*They would follow Cathy's advice; they would give the baby water so that the throat would not be dry. The baby must also drink water so that the throat is not burned.*

*(R3: Mother of unmarried FTM 15–19 years)*

FTMs shared that high temperatures caused the child to perspire and this was an indication that the child needed something cool to replenish water that had been lost. One mother shared:

*Water is life. When we sweat, we lose water! Because of this, you have to drink to revitalize yourself. The baby needs to drink water. If you have to wait six months, your baby's health may deteriorate. You do not know what he has in the body, but by giving him water, you allow him to eliminate some things even diseases. In addition, breast milk is hot, it's good to give water to the baby because it's good for his health.*

*(R11: Unmarried FTM 15–19 years)*

##### Social influence

Participants reported that social influence was a deciding factor in providing water to the newborn. Many male partners and FTMs shared that mothers gave water because of a family member’s or friend’s previous experience with raising children.

*She will follow her mom because her mother has a lot of experience and she has already done these things, so she will give water to her child after five days.*

*(R2: Male partner of unmarried FTM 15–19 years)*

*She will give water to the child because her mom and friend told her the same thing. She will accept it this time and give the baby some water in addition to breast milk.*

*(R2: Mother of unmarried FTM 20–24 years)*

The perceived reaction of and respect for the FTM’s mother or mother-in-law could also influence the decision to provide water. One participant shared:

*It depends. She may or may not refuse, but many will give water, because "libele eshofaka" (the breast of the mother heats up when the temperature outside increases). Out of respect for her mother, Marie will give water to her child.*

*(R3: Mother-in-law of FTM 20–24 years)*

In some cases, participants suggested that FTMs may lack the agency in decision making regarding exclusive breastfeeding because of their age. A woman’s desire to exert power and control over her daughter/daughter in law could lead to a disregard of infant feeding recommendations by health professionals. One participant shared:

*She must do what I say. She may say, “mom, listen to what was said in the hospital.” Well, that is not my problem. She will do what I say, even if I say give the child foufou [a common staple made from cassava and corn flour] or something else, she will obey.*

*(R11: Mother-in-law of FTM 15–19 years)*

*Personally, I don’t say what the providers say. When we wash the child, he tries to drink water. She would tell me what the provider said. When we bathe the child, my daughter must follow what I say. If she does not do that, I get mean. If I think about things like that…. I get upset.*

*(R10: Mother-in-law of FTM 15–19 years)*

#### Support for exclusive breastfeeding

While many participants supported the provision of water to newborns, there were some who supported exclusive breastfeeding. This was particularly the case for most mothers and mothers-in-law of FTMs, as well as for a minority of FTMs and male partners. Supporters believed that Marie was not given good advice and other FTMs would exercise personal agency in breastfeeding and not give water to their baby. They shared that these FTMs would be knowledgeable about the detrimental health consequences and would have been counseled by a health provider.

*She is not going to obey because she is the one who gave birth to the child. Marie gave birth to her child, so she will follow what she was told to the hospital, not give water to the child for six months.*

*(R1: Mother-in-law of FTM 15–19 years)*

*I put myself in the place of Marie. If the doctor told me to feed my baby only breast milk, that's what I will do and for six months. And if the doctor tells me that after six months, I can give water, I'll do it.*

*(R4: Unmarried FTM 20–24 years)*

*I say that most new mothers age 15-24 would not follow Cathy's advice, and would not give water to the child of that age. Based on what I know, you have to breastfeed the child until the age of 6 months before giving him water. Never give water to the child because that can cause diseases.*

*(R2: Male partner married to FTM 15–19 years)*

Some mothers and mothers-in-law openly acknowledged that they used to give their own newborns water, but times have changed. They suggested that their daughters had been empowered and were vocal about their breastfeeding decisions. Thus, they (mothers and mother-in-law) must respect the FTM’s autonomy.

*If she is a smart girl, even if her mother tells her to give water to the child because it's hot, she must not follow her mother's instructions. She must remind herself that the things done in her mom’s time are not the same as today.*

*(R7: Mother-in-law of FTM 20–24 years)*

Although there was agreement among participants that mothers should not give water to their newborns, the age at which participants felt that water should be introduced varied. Most frequently, participants mentioned 6 months as the appropriate time to start giving newborns water, but there were some who shared that water intake could begin as early as 2 months of age.

*Normally, when the child is a newborn, it is not given water. The child is given breastmilk. We could give water after three months. You can then start giving the baby water little by little until six months for him to start eating. Marie’s mother did not give good advice. The child is too small and should not be given water. They only need breast milk, and for milk to come out well, it requires that the mother eats well. The child is healthy when they drink the mother’s milk.*

*(R3: Male partner married to FTM 20–24 years)*

*I agree with the decision Marie took; if it was the 5th month, I would accept it ... For other women, once they are offered this, they may wait for the child to be two months old before they will give water. We were taught at ANC that it is in the 6th month that water should be given, but sometimes we do not respect that, we do it after 2 or 3 months.*

*(R8: Married FTM 15–19 years)*

Some participants, mainly male partners, were neutral because they felt that the mother of the baby would need to consult a health provider prior to making the decision.

*She will have to go back to the hospital so that the doctors give advice about breastfeeding and providing water to the child in addition to breastmilk*

*(R9: Male partner married to FTM 15–19 years)*

### Normative expectations regarding breastfeeding

Overall, the majority of FGD participants expected Marie to take Cathy and her mother’s advice and give the newborn water. All FTMs participating in the study had this expectation, regardless of age and marital status. Male partners, mothers and mothers-in-law, on the other hand, had mixed expectations. While most did not provide an explanation, a few suggested that Cathy would give water because water improved the health and wellbeing of the newborn and Marie’s mother was speaking from experience, and because of peer influence.

*They expect Marie to change her decision and give water to the child.*

*(R4: Married FTM 15–19 years)*

*They expect her to do what the mother told her.*

*(R5: Male partner of unmarried FTM 20–24 years)*

*They would expect Marie to apply this advice and give water to the baby. Breast milk heats the stomach, so the baby should also take water.*

*(R7: Mother of unmarried FTM 15–19 years)*

*The others would expect Marie to give her child water, but if she does not give, they will say, she is proud, she only follows doctors, does not even give water to their children.*

*(R1: Mother-in-law of FTM 20–24 years)*

A minority of participants did not expect Marie to change her mind and give the newborn water. Although no FTM had this expectation, the other participants expressed this opinion. Some mothers and mothers-in-law alluded to the importance of the advice provided during ANC or by a provider and others explained that the child was Marie’s responsibility, alluding to the importance of personal agency in breastfeeding decisions.

*Marie is not going to give water to this child, because he is her child.*

*(R6: Mother-in-law of FTM 15–19 years)*

*If I were in the place of Marie, I will not accept the advice of other people because after giving the child water, other complications can occur. Doctors asked us to give only [breast]milk to the child up to six months. They have to respect it.*

*(R3: Mother of unmarried FTM 15–19 years)*

A few male partners, mothers and mothers-in-law also recognized the potential influence of health providers in Marie’s decision. These participants expected Marie to rely on the providers’ advice given during an ANC visit or, alternatively, Marie would seek advice from providers before making a decision.

*Cathy and most new mothers will expect Marie to wait until the time she has been advised by the doctors and midwives to give water to her child. They [Cathy and most new mothers] do not have the right to force Mary to give water to her child.*

*(R3: Male partner married to FTM 15–19 years)*

### Rewards and punishments related to exclusive breastfeeding

Most respondents, including FTMs, reported that the practice of exclusive breastfeeding is met with both positive and negative sanctions from peers and community members. However, participants tended to agree that negative sanctions or punishments were more likely to ensue.

#### Punishments

Verbal abuse and social isolation were the most common forms of punishment young FTMs faced for practicing exclusive breastfeeding despite their mothers’ and peers’ belief that they should give water to the newborn baby.

##### Verbal abuse

Name-calling (“bad person”, stubborn), insults, and slandering were the most reported form of verbal abuse. This theme was predominant among older, unmarried or no longer married FTMs, and their mothers and male partners. Verbal abuse was motivated by the perceived negative impact the young FTM’s decision to exclusively breastfeed would have on her baby, and on her relationship with her mother and peers. Concerns over possible detrimental effects of exclusive breastfeeding on babies were mainly driven by the fact that breastfeeding competes with water consumption by the baby.

*They will say that Marie [FTM] does not listen to the advice. She is bad, she gave birth and she does not want to give water to her child.*

*(R1: Male partner married to FTM 20–24 years)*

*Cathy and most FTM would say that Marie is stubborn, she wants to dehydrate her child because she doesn’t want to give her water in addition to breastmilk.*

*(R12: Unmarried FTM 20–24 years)*

On the other hand, respondents pointed that the FTM would be called names because she ignored the advice given to her by her mother and her friend, both of whom were seen as trusted and more experienced figures.

*They will say that Marie is a bad girl, she did not want to listen the advice given to her by Cathy and her mother, and yet that was good advice.*

*(R10: Mother of unmarried FTM 15–19 years)*

*They will criticize her a lot. They will say how is that girl? Her own mother spoke to her and she does not want to listen. Even us, as many as we are, she still doesn’t want to listen. She will be slandered.*

*(R11: Mother-in-law of FTM 15–19 years)*

*They will say that Marie is stubborn, she is not informed about all those things and why doesn’t she want to listen to people who are more experienced?*

*(R6: Married FTM 15–19 years)*

*I think Marie is very stubborn, her mother gave her an advice and she did not want to take it in consideration. Her friend gave her the same advice and she reacted the same way. She is stubborn.*

*(R9: Mother of unmarried FTM 20–24 years)*

Finally, the community’s resistance to the practice of exclusive breastfeeding by young FTMs appeared to originate, at least partially, from the lack of trust in healthcare providers. One FTM reported:

*Cathy and most first-time mothers would say that Marie is naïve, the nurses at the health center are lying to her, and she accepts these kinds of things.*

*(R4: Unmarred FTM 20–24 years)*

##### Social isolation

The FTM could be socially isolated because of her decision to practice exclusive breastfeeding. Isolation was most reported by unmarred FTM age 20–24 years and the male partners of unmarried FTMs age15–19 years. In most instances, participants reported that FTMs who decided to practice exclusive breastfeeding would be “left alone”. This punishment could include any type of isolation, but in this context, it often referred to the avoidance of discussing any topic related to the baby with the FTM.

*If Marie maintains her position, they will leave her alone. If they insist and she refuses, they will leave her alone. They can say that Marie is making her baby suffer, and they can also say that it [giving water to the baby] will make her life easier, rather than giving only breastmilk to the baby.*

*(R3: Married FTM 15–19 years)*

*I will leave her alone since she prefers to breastfeed her baby up to 6 months. I cannot oblige her to give water to her baby. I cannot judge her for that. But it hurts when you give a kind-hearted advice to a friend who refuses to accept it.*

*(R1: Unmarried FTM 20–24 years)*

*They will say because you have refused the advice, we will not be interested in you anymore.*

*(R11: Male partner of unmarried FTM 15–19 years)*

In some extreme cases, respondents suggested that people should distance themselves from the FTM, so she could suffer the consequences of her decision if something [bad] happens to the baby because of her refusal to give the child water.

*Cathy and most first-time mothers aged 15-24 years would say to let her practice exclusive breastfeeding for six months, all the consequences will teach her. Leave her because she does not want to listen our advice.*

*(R1: Male partner of married FTM 15–19 years)*

*Cathy and most first-time mothers will say Marie is stubborn, she does not listen [to our advices]. She will see all the consequences happening to her as she does not want to listen.*

*(R6: Unmarried FTM 20–24 years)*

In the same order, many people reported that an FTM who was not compliant with socially accepted values and practices around breastfeeding would be held responsible for anything that happened to her baby. However, it was not clear whether a change in the FTM’s position could revert these sanctions.

*They will condemn her; they will say if something happened to the child it will be on you because you chose to do what they told you at the hospital.*

*(R1: Male partner of unmarried FTM 15–19 years)*

*They will say it is her problem. She will be the only responsible if a health problem happens to her baby.*

*(R11: Married FTM 20–24)*

Finally, the FTM would be left alone because she would be perceived as unfit for her community. Resisting trusted figures and going against widely accepted practices is perceived as something from a different culture. The following declaration encapsulated this viewpoint:

*Friends will say she is boastful; she wants to raise her child like a white person.*

*(R8: Mother-in-law of FTM 15–19 years)*

In this context, ‘white person’ referred to foreign culture.

A small group of respondents reported neither form of sanction. Instead, they believed that the decision of the FTM to practice exclusive breastfeeding would be challenged by her friends and other people in the community, the goal being to convince her of the benefits of giving water to the baby. The mothers of unmarried FTMs 15–19 years were the only participants to report it.

*The other FTMs will say that Marie is taking a bad decision, she should give water to her baby to allow a better digestion, otherwise the baby will not be able to breastfeed correctly because she will have milk in her stomach.*

*(R9: Mother of unmarried FTM 15–19 years)*

#### Rewards

A few respondents praised exclusive breastfeeding practices by young FTMs. They associated the practice of exclusive breastfeeding with the observance of medical recommendations, which could guarantee good health for the mother and baby. Moreover, some pointed that FTMs who chose to breastfeed exclusively demonstrated a commitment to providing what they believed was best for their baby.

### Influence of social network (conditional preferences)

We investigated whether young FTMs’ position regarding exclusive breastfeeding was conditional on the opinions and the reactions of their peers. Across all the groups, respondents shared that peers had enough influence to make the FTM change her decision. Although this viewpoint was preponderant, it was not unanimous. Some FTMs and male partners, mothers, and mothers-in-law of FTMs aged 15–19 years old believed that the FTM would not change her mind based on her peers’ opinions and reactions.

#### Factors enabling peers influence

A potential change in breastfeeding practices based on peers’ reactions did not appear to be motivated by a real change in the FTM’s opinion about breastfeeding. Instead, social pressure appeared to play a major role.

##### Social pressure

The FTM would be pressured to adopt socially accepted breastfeeding practices through various mechanisms, the most important of which is through constant exposure to interpersonal communication messages from members of her social network, promoting the benefits of water for newborns. In fact, given how widespread this belief is in Kinshasa, the FTM would likely be exposed to the same message outside her family and friends circles (neighbors, church members, etc.). In this case, the message would be reinforced and could eventually cause the FTM to change her mind.

*She will think about it. When she realizes that many people are telling her the same thing, she will do it later.*

*(R7: Male partner of unmarried FTM 15–19 years)*

*The opinions of Marie’s friends can make her change her mind. If they tell her that it is not good for the baby to take only breastmilk, and if they insist, she will change her position.*

*(R1: Mother of unmarried FTM 20–24 years)*

In addition to social pressure, FTMs could also revert from breastfeeding their baby exclusively because of their trust in their peers, fear of punishment, lack of experience, fear of consequences, or because of no clear reason.

##### Trust in peers

Trust seems to be a major factor in FTMs’ susceptibility to peer influence, denoting the role of formal and informal social networks in important health-related decisions among this population. This theme was prevalent among FTMs participating in the study.

*Yes, because we young girls trust our friends too much, even if what they tell us is not true, we will continue to trust them.*

*(R8: Unmarried FTM 20–24 years)*

*“Yes, because friends have a great influence on us young girls. I would believe everything a friend tells me because I trust them”*

*(R9: Unmarried FTM 20–24 years)*

##### Fear of punishment

The analysis suggested that punishment had a long-term effect on the FTM. Usually, FTMs would amend their position when they started feeling the effects of social exclusion or when they realized how much people “talked about them.” In both cases, the change would be motivated by the desire to be reintegrated in their social circles.

*When she realizes that people are abandoning her because she did not want to listen to them, she will reconsider her position and give water to her baby.*

*(R1: Male partner of unmarried FTM 15–19 years)*

##### Lack of experience with childrearing

A change in breastfeeding practices would be also determined by how FTMs compared themselves to their influencers in relation to childrearing experience. The more the FTM perceived others as being more experienced, the more susceptible would she be to change her breastfeeding practices and adopting behaviors that were more perceived to be more socially accepted.

*If she listens to her friends’ opinions and if she is not strong, she will cede and give water to her child. She will say that it is not a big deal since many have given water to their children and nothing happened. She will give water based on what she learns from her friends.*

*(R2: Mother-in-law of FTM 20–24 years)*

##### Fear of consequences

The perceived negative consequences of exclusive breastfeeding, especially the consequences of water deprivation on her baby’s health, could weaken the FTM’s motivation to practice exclusive breastfeeding.

##### Lack of clear reasons

Some participants felt that after discussing breastfeeding practices with their peers, FTMs could just think about it and change their minds. Although this was one of the most prominent beliefs, the lack of appropriate probes did not allow us to understand the mechanisms that would motivate a change in breastfeeding practices under such circumstances.

#### Protective factors against peer influence

Despite their susceptibility to social pressure, it appeared that an FTM’s understanding of medical recommendations about and benefits of exclusive breastfeeding could act as a protective factor against negative peer influence.

*Yes, the reactions of Marie’s friends could make her change her mind about exclusive breastfeeding practices […] she can be negatively influenced by her friends, especially if she did not understand quite clearly the advice given to her by the midwives, and if Marie does not really trust the midwives she can change her mind.*

*(R2: Male partner of married FTM 15–19 years)*

*She will not change her mind if she has good knowledge about it [exclusive breastfeeding].*

*(R11: Married FTM 20–24 years)*

*Her position will not change because only breastmilk provides proteins. Does water provide proteins?*

*(R6: FTM 20–24 years no longer married)*

Similarly, trust in healthcare providers would reduce FTMs’ susceptibility to negative peer influence on exclusive breastfeeding.

*She will not follow her friends; she will obey what the physicians told her to do. Her friends can talk but she will not cede because if her child becomes sick today none of her friends will contribute to his health care, she will have to provide for everything.*

*(R3: Mother-in-law of FTM 20–24 years)*

## Discussion

This study showed that overall, community norms in Kinshasa were not supportive of exclusive breastfeeding. Many participants shared that Marie and most new mothers aged 15–24 did not follow the practice of exclusively breastfeeding their newborns and they expected Marie to accept Cathy and her mother’s advice to give the newborn water. Reasons included the desire to please key members of their social networks, specifically their mothers and friends by doing what these influencers expected or preferred them to do; FTMs own lack of experience with infant feeding; and trust placed in their mothers and friends. Even though it is well established that human milk is the ideal nourishment for infants’ survival, growth and development, some participants subscribed to myths that water was also necessary for the newborn’s growth as human milk was perceived to be hot and water as vital to life. Many mothers who refused to give babies water in the first 6 months were felt to face negative sanctions such as name-calling and mockery; participants shared that FTMs would provide water to newborns to avoid these sanctions.

Most participants noted that when faced with social pressure to give babies water during the first 6 months of life in addition to breastmilk, the FTM would be motivated to imitate what other women in her social environment do. The reference groups for decisions pertaining to exclusive breastfeeding included health care workers, friends, mothers, and mothers-in-law, and these groups were perceived as having conflicting norms regarding exclusive breastfeeding. While many male partners of married FTMs and mothers-in-law felt that a new mother would be likely to adhere to the recommendations of health workers to exclusively breastfeed her baby for 6 months or seek their advice if unsure, male partners and mothers of unmarried FTMs felt that the latter were obligated to listen to their mother’s advice even if such advice included giving the baby water in addition to breastmilk during the first 6 months of life.

Our study adds to the literature on exclusive breastfeeding in Kinshasa by shedding light on the social norms and barriers around the practice. Previous studies in the DRC and other parts of sub-Saharan Africa partly support findings pertaining to women’s own lack of experience in infant feeding [8–11, 21–23]. For example, in a quantitative analysis, Babakazo et al. found that discontinuation of exclusive breastfeeding before 6 months was significantly associated with lack of confidence in the ability to breastfeed, low level of breastfeeding knowledge, as well as with having no plan on the duration of breastfeeding and breastfeeding problems during the first week following childbirth [9]. A quantitative study in rural Kenya, found that a more positive perception of the acceptability of exclusive breastfeeding by key influencers was associated with significantly lower risks of premature cessation of exclusive breastfeeding [22]. The latter study also found that mother’s beliefs about the impact of exclusive breastfeeding on the mother’s health, physical appearance and ability to engage in other activities had the strongest relationship with premature cessation of exclusive breastfeeding [22].

In Uganda and Rwanda, studies suggest that the negative influence of female family members, such as sisters, mothers and mothers-in-law, leads to suboptimal feeding practices for the baby [24, 25]. The study in Uganda also links the non-observance of exclusive breastfeeding to the belief that breastmilk is not enough to satiate the baby and the perception that breastfeeding is indicative of poverty [24]. Similar findings have also been documented in Asia, Latin America, and the Caribbean [26, 27]. While our study did not capture the perceived advantages of exclusive breastfeeding for the mother’s health, the FGDs revealed that the perception of exclusive breastfeeding as having positive benefits for child health occurred among participants who approved of Marie’s insistence to continue exclusive breastfeeding despite negative social pressure.

Exclusive breastfeeding for 6 months and early initiation of breastfeeding are a central part of the “Every Woman Every Child” global strategy and are essential for achieving Sustainable Development Goal targets on child survival, health and nutrition. To achieve these targets, it is essential for programs to develop targeted messages to positively influence social norms and reverse the practice of giving water to the baby before the age of 6 months. Exclusive breastfeeding education messages should target the FTMs and multiple influencers of FTM’s exclusive breastfeeding decision, including the FTM’s male partner, mother, family members, and friends. Health communication programs may be more effective if they engage the entire community in a collective discussion about the positive effects of exclusive breastfeeding for 6 months in order to create a positive social environment for adherence to the practice. A critical component of any communication strategy is to correct existing misperceptions about exclusive breastfeeding and improve FTM’s self-efficacy to withstand social pressure to not breastfeed their babies exclusively for 6 months. Self-efficacy can be strengthened through participation in support groups with peers who exclusively breastfeed. The support groups provide expectant and new mothers with a space to build positive peer networks and the skills necessary to exclusively breastfeed. Moreover, it can increase their perceived prevalence of breastfeeding in the community when deciding whether or not to exclusively breastfeed.

Providing rewards to mothers who practice exclusive breastfeeding can counteract the strong negative social sanctions against exclusive breastfeeding - for example, recognizing mothers who exclusively breastfeed their newborns in the community or at the health center. This reward celebrates and promotes exclusive breastfeeding decisions. While rewards and the various strategies discussed can be effective in promoting exclusive breastfeeding, some strategies may have potential negative impacts for some mothers. Thus, the choice of the intervention strategy should be determined by the local context and the potential risks of the strategy. In addition, interventions should use multiple strategies as they have been found to have a more significant impact than those with individual approaches [28, 29].

Our findings also suggest a need to improve the relationship and trust between clinic-based health providers and the community at large. Although our research did not shed much light on male partners’ role in exclusive breastfeeding support, it is important for health communication programs and health professionals to improve fathers’ knowledge about the benefits of exclusive breastfeeding during the prenatal period and at delivery, and to encourage them to support their partners to adhere to the recommended duration of the practice. Research is needed on fathers’ attitudes towards exclusive breastfeeding and the best strategies for paternal involvement in exclusive breastfeeding education and promotion.

The study has some limitations. First, the sample selection was purposive, and our findings are not generalizable to a wider population. Second, most of our focus group participants were unemployed. FTMs, male partners, and mothers and mothers-in-law who work are difficult to access, and biases may arise if those who are employed perceive different norms, barriers, facilitators and key influencers of exclusive breastfeeding. Residence in Kinshasa for a year was an inclusion criterion for participation in the study. However, it limited our understanding of exclusive breastfeeding norms and practices among recent migrants to Kinshasa, which is important since the city is among the top five most rapidly growing urban areas in sub-Saharan Africa. Our qualitative approach and use of a vignette did enable us to gain a clearer understanding of the social processes that govern FTMs’ decisions to initiate exclusive breastfeeding and when to discontinue it.

## Conclusion

In Kinshasa, social norms were not supportive of exclusive breastfeeding, and the provision of water to babies under the age of 6 months was widely accepted. We found that participants perceived prevalence of exclusively breastfeeding among FTMs in the community was low and this tended to influence whether or not FTMs practiced exclusive breastfeeding. We also found that injunctive norms against exclusive breastfeeding were strong, with most participants perceiving the mothers and friends of FTMs to disapprove of exclusive breastfeeding practices. In addition, we identified that there were strong normative expectations that water be given to newborns in addition to breastmilk, and that there were strong negative social sanctions against exclusive breastfeeding. However, our findings suggested that FTM’s must have high levels of perceived self-efficacy to withstand social pressure to not exclusively breastfeed. Addressing negative social attitudes has the potential to increase the prevalence and duration of exclusive breastfeeding. Efforts should focus not only on the FTM but also on those who influence infant feeding decisions, such as FTMs’ mothers, mothers-in-law, male partners, and friends.

## Supplementary information

**Additional file 1.**

## Data Availability

The transcripts from the study are available (in French) from the corresponding author on reasonable request.

## Abbreviations

CMO: Chief Medical Officers
DRC: Democratic Republic of Congo
FGD: Focus group discussion
FTM: First-time mother
HIV: Human Immunodeficiency
UNICEF: United Nations Children’s Fund
WHO: World Health Organization
