# Supplementary material for: Insights on exclusive breastfeeding norms in Kinshasa: findings from a qualitative study
Source: BMC Pregnancy Childbirth. 2020 Oct 6;20:586. doi: 10.1186/s12884-020-03273-4 (PMC7539451; doi:10.1186/s12884-020-03273-4)
Supplement: Supplementary file 1 — Additional file 1. [file 12884_2020_3273_MOESM1_ESM.docx]

**FORMATIVE RESEARCH FOR THE MOMENTUM PROJECT IN KINSHASA, THE DEMOCRATIC REPUBLIC OF CONGO**

**Focus Group Discussion**

**GENDER NORMS AND MATERNAL AND NEWBORN HEALTH**

**Breastfeeding section**

**August 7, 2017**

**OBJECTIVE: IDENTIFY HOW DECISIONS ABOUT BREASTFEEDING ARE MADE**

Now, let’s talk about breastfeeding. I will tell you a story about Marie who is a first-time mother and is 18 years old. Marie gave birth to a healthy baby boy five days ago. Marie is practicing exclusive breastfeeding.  Marie’s friend, Cathy, comes to visit her. They are about the same age. Cathy strongly suggests to Marie that she should give her baby water in addition to breast milk. Cathy argues that Marie should give her baby water to drink because it is too hot and because water is needed for proper digestion of breast milk. Marie’s mother who has been listening to the conversation supports Cathy’s arguments. Marie’s mother is happy that Cathy has given Marie this advice because Marie has not been listening to her mother’s advice.

1. What would most 15-24-year-old first-time mothers in Marie’s situation do in this situation? Would they give the newborn water in addition to breastmilk?
2. What would Cathy and most other first-time mothers expect Marie to do in this situation?
3. If Marie decided that in spite of what Cathy says she would exclusively breastfeed her baby for the first six months, what would Cathy and most other first-time mothers who are 15-24 years old say about Marie’s decision?
4. Would the opinions and reactions of her friends make Marie change her mind about practicing exclusive breastfeeding for six months?
5. Are there other people whose opinions and reactions would make Marie give her newborn water in addition to breastmilk?
6. Are there any circumstances where it would be considered more or less acceptable for Marie to breastfeed her baby exclusively for six months?
